# Single-particle properties of topological Wannier excitons in bismuth chalcogenide nanosheets

**DOI:** 10.1038/s41598-023-32740-z

**Published:** 2023-04-18

**Authors:** Lucas Maisel Licerán, Francisco García Flórez, Laurens D. A. Siebbeles, Henk T. C. Stoof

**Affiliations:** 1 Institute for Theoretical Physics and Center for Extreme Matter and Emergent Phenomena, Utrecht University, Princetonplein 5, 3584CC Utrecht, The Netherlands; 2 Optoelectronic Materials Section, Department of Chemical Engineering, Delft University of Technology, Van der Maasweg 9, 2629HZ Delft, The Netherlands

**Keywords:** Topological insulators, Two-dimensional materials, Quantum Hall, Two-dimensional materials, Electronic properties and materials

## Abstract

We analyze the topology, dispersion, and optical selection rules of bulk Wannier excitons in nanosheets of Bi_2_Se_3_, a topological insulator in the family of the bismuth chalcogenides. Our main finding is that excitons also inherit the topology of the electronic bands, quantified by the skyrmion winding numbers of the constituent electron and hole pseudospins as a function of the total exciton momentum. The excitonic bands are found to be strongly indirect due to the band inversion of the underlying single-particle model. At zero total momentum, we predict that the *s*-wave and *d*-wave states of two exciton families are selectively bright under left- or right-circularly polarized light. We furthermore show that every *s*-wave exciton state consists of a quartet with a degenerate and quadratically dispersing nonchiral doublet, and a chiral doublet with one linearly dispersing mode as in transition metal dichalcogenides. Finally, we discuss the potential existence of topological edge states of chiral excitons arising from the bulk-boundary correspondence.

## Introduction

Three-dimensional topological insulators, and all other topological materials for that matter, are presently receiving much attention because of their excellent prospects for energy-efficient electronics, (pseudo)spintronics devices, and quantum information processing^1–18^. Prototypical examples of three-dimensional topological insulators are the bismuth chalcogenides Bi$_2$Se$_3$ and Bi$_2$Te$_3$. Since in linear response these materials are ideally conducting only due to the presence of massless Dirac fermions on their surface, most experiments with topological insulators have focused on these unusual topologically protected surface states. However, the situation changes dramatically upon photoexcitation, as excitons and unbound charges may be produced in the bulk with a topologically nontrivial band structure. Consequently, it is important for the understanding of light-matter interactions to investigate also the bulk properties of topological insulators and in particular the precise topological nature of the excitons, whose presence or absence is crucial in optoelectronic devices such as lasers^19–22^, light-emitting diodes^23–25^, and photovoltaic cells^26–29^. In the context of quantum information processing, a particularly interesting question is if the exciton topology is transferred to the quantum state of the photons emitted via photo- or electro-luminescence^30^. Apart from such applications, the many-body physics of topological excitons is thought to be very exciting and accessible experimentally by well-established pump-probe techniques. Important examples of interesting many-body states are the topological excitonic insulator^31–35^ and a Bose-Einstein condensate of topological excitons or possibly even biexcitons^36^.

In recent years, therefore, there has been an increasing interest in the study of excitons formed in topological insulators. Semiclassically and within the effective-mass approximation, a pioneering approach has been to introduce Berry-phase corrections to the electron-hole interaction due to the topological band structure and determine the exciton energy spectrum^37–42^. The approach presented in these works mostly focuses on the case of a total exciton momentum $\varvec{Q} = \varvec{0}$, which is sufficient for studying optically active excitons, but not enough to obtain their global topological properties. The latter has been achieved for Frenkel excitons within a Hubbard-like model with on-site Coulomb interactions^43^. However, because of the insulating nature of the bulk of bismuth chalcogenide nanosheets and their geometry, which leads to a lower-than-bulk dielectric constant due to the surrounding medium, we expect in these materials long-ranged electron-hole interactions allowing for the formation of so-called topological Wannier excitons^44,45^. In this article we therefore study the concrete example of (quasi-)two-dimensional bulk excitons in Bi_2_Se_3_ nanosheets.

## Results

### Band structure of Bi_2_Se_3_ nanosheets

**Figure 1:**
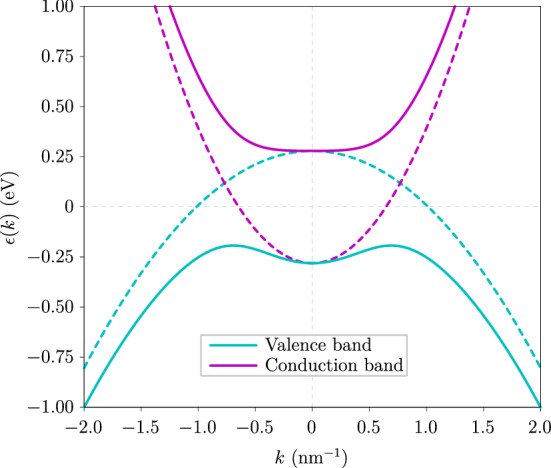
Band structure of two-dimensional Bi$_2$Se$_3$ around the $\Gamma$ point. Both the conduction bands and the valence bands are shown in solid lines. Each is two-fold degenerate, and due to spin-orbit coupling features a band inversion and an avoided crossing of the corresponding uncoupled states depicted with dashed lines. The dispersions are isotropic in the $\varvec{k} \varvec{\cdot } \varvec{p}$ approximation we are using.

Because we are only interested in the physics around the $\Gamma$ point, we restrict ourselves to the bands closest to the Fermi surface. For all numerical purposes we choose a nanosheet thickness of 6 nm, which approximately corresponds to 6 quintuple layers (QLs). This is sufficiently large to preserve the nontrivial bulk topology, but also sufficiently small for the problem to be mainly regarded as two-dimensional. The effective Hamiltonian governing the physics around the $\Gamma$ point in Bi_2_Se_3_ nanosheets is equivalent to the BHZ Hamiltonian for the quantum spin Hall effect^46^. As a consequence of spin-orbit coupling it features a band inversion around the $\Gamma$ point as illustrated in Fig. 1. As a combined effect of the time-reversal and inversion symmetries of the system, the conduction and valence bands are each two-fold degenerate. We label each band by an index denoted as the spin-orbit parity and obtain four eigenstates $| \chi ^{\mu }_{\varvec{k}} \rangle$, where $\mu \in \{c,v\} \times \{+,-\}$.

### Topological exciton eigenstates

**Figure 2:**
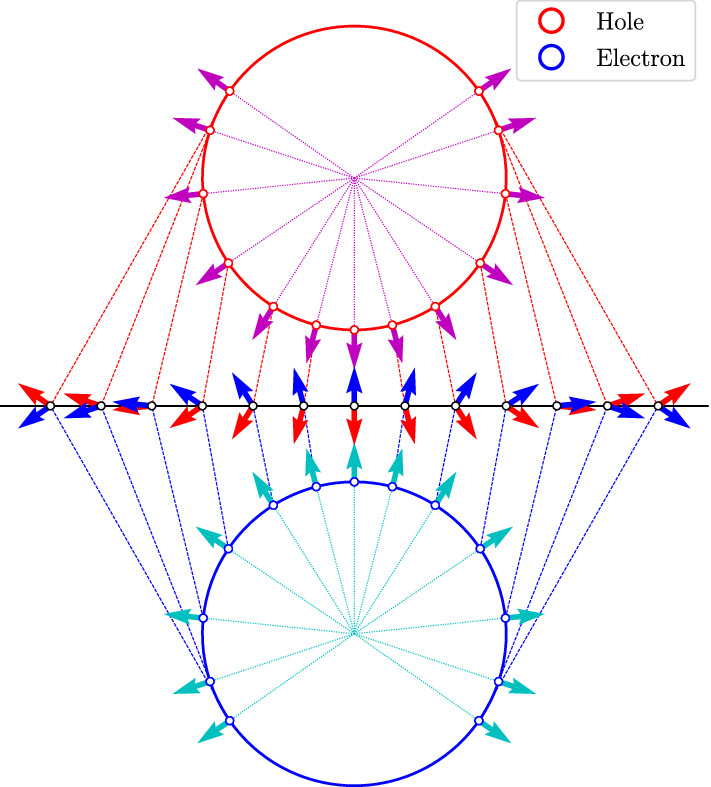
Momentum-space skyrmion textures of an electron and a hole. This idealized illustration corresponds to the configuration in a weakly bound exciton. Due to rotational symmetry the two circles actually represent only a radial slice of the two spheres onto which the complete momentum plane is mapped with a unit winding number. The south (north) pole of the top (bottom) sphere corresponds to the origin $Q = 0$, whereas the opposite pole corresponds to $Q \rightarrow \infty$. Note that in a normal semiconductor like CdSe the pseudospins always point in the same direction, independent of *Q*, and thus have a zero winding number.

The four different spin-orbit-parity combinations for an electron and a hole give four different exciton families $| \varvec{Q};s,t \rangle$ that generalize the singlet and triplet states in normal semiconductors. Here, *s* and *t* are the spin-orbit parities of the bands where the electron and the hole are located, respectively, and $\varvec{Q} \equiv (Q_{x}, Q_{y})$ is the total in-plane exciton momentum. In the absence of an exchange interaction, these states diagonalize the two-body Hamiltonian and have a well-defined Chern number equal to $\mathcal {C}_{s,t} = s + t$. Hence, in this idealized case there are two topologically nontrivial exciton states, characterized by a nontrivial winding of the momentum-space pseudospin texture $\varvec{\Gamma }_{e,h}(\varvec{Q})$ (defined precisely in the “Methods” section). As illustrated in Fig. 2, the pseudospin texture gives an intuitive picture of the nontrivial topology of this basis of exciton states, as it allows us to visualize the total Chern number of each basis element as a combination of two winding numbers by looking at the path of the electron (hole) pseudospin from pointing upwards (downwards) at the origin $Q = 0$ to pointing downwards (upwards) at $Q \rightarrow \infty$, represented as the circles in Fig. 2. Thus, the pseudospin texture of each constituent particle can be seen as a skyrmion, so that the topological exciton is represented by a double skyrmion texture.

**Figure 3:**
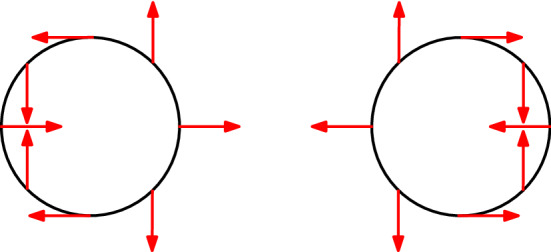
Behavior of the spin-orbit-parity pseudospin around the origin of momentum. As the polar angle $\phi _{\varvec{Q}}$ varies from 0 to $2 \pi$, the pseudospin $\varvec{\sigma }$ of the states $| \varvec{Q}; 2_{+} \rangle$ (left) and $| \varvec{Q}; 2_{-} \rangle$ (right) winds around twice, as its orientation is coupled to that of the total exciton momentum.

Including the exchange interaction couples $| \varvec{Q};+,+ \rangle$ with $| \varvec{Q};-,- \rangle$, and this idealized picture complicates somewhat. Analyzing the effective two-dimensional interaction between electrons and holes, we find that the exciton eigenstates split into two doublets as

1 $$\begin{aligned} | \varvec{Q};0_\pm \rangle&\equiv \frac{1}{\sqrt{2}} \Big ( | \varvec{Q};+,- \rangle \pm | \varvec{Q};-,+ \rangle \Big ) ~, \end{aligned}$$

2 $$\begin{aligned} | \varvec{Q};2_\pm \rangle&\equiv \frac{1}{\sqrt{2}} \Big ( e^{- i \phi _{\varvec{Q}}} | \varvec{Q};+,+ \rangle \pm e^{i \phi _{\varvec{Q}}} | \varvec{Q};-,- \rangle \Big ) ~, \end{aligned}$$

with $\phi _{\varvec{Q}}$ the polar angle of $\varvec{Q}$ with respect to the *x*-axis. The wave functions contained in $| \varvec{Q};+,- \rangle$ and $| \varvec{Q};-,+ \rangle$ are related by complex conjugation, and so are those contained in $| \varvec{Q};+,+ \rangle$ and $| \varvec{Q};-,- \rangle$. Due to the combined phase factors $e^{\pm i \phi _{\varvec{Q}}}$, the members of the doublet $| \varvec{Q}; 2_{\pm } \rangle$ have chirality two, a signature of the nonzero Berry curvature within the nanosheet. This chirality can be understood via the introduction of a spin-orbit-parity pseudospin operator $\varvec{\sigma } \equiv (\sigma _{x}, \sigma _{y}, \sigma _{z})$, where $\sigma _{i}$ are the Pauli matrices acting on the space spanned by $| \varvec{Q};+,+ \rangle$ and $| \varvec{Q};-,- \rangle$. Its expected value for the states $| \varvec{Q}; 2_{\pm } \rangle$ is

3 $$\begin{aligned} {\langle \varvec{\sigma } \rangle }_{2_{\pm }} = \pm \cos (2 \phi _{\varvec{Q}}) \, \hat{\varvec{x}} \pm \sin (2 \phi _{\varvec{Q}}) \, \hat{\varvec{y}} ~, \end{aligned}$$

and its behavior on the momentum plane is sketched in Fig. 3. We see that it has a winding number of 2 around $\varvec{Q} = \varvec{0}$, analogously to the valley pseudospin in Ref.^47^. We mention, in passing, that the chiral excitons considered here are of different nature from those observed in the experiment of Kung et al.^30^ Indeed, the latter result from high-energy transitions between massive holes and massless Dirac electrons on the surface, whereas the ones in this work arise from long-wavelength transitions between bulk bands near the Fermi level.

We may further label the different individual states in $| \varvec{Q};0_{\pm } \rangle$ by a principal quantum number $n \in \{0,1,2,\dots \}$ and by their at $\varvec{Q} = \varvec{0}$ well-defined relative angular momentum $m \in \{-n,\dots ,0,\dots ,n\}$, introducing the notation $| \varvec{Q};0_{\pm };n, m \rangle$. On the other hand, the individual states in $| \varvec{Q};2_{\pm } \rangle$ are similarly labeled by the at $\varvec{Q} = \varvec{0}$ well-defined angular momentum *m* of the $| \varvec{Q};+,+ \rangle$ component in their linear combination.

### Exciton dispersion relations and wave functions

**Figure 4:**
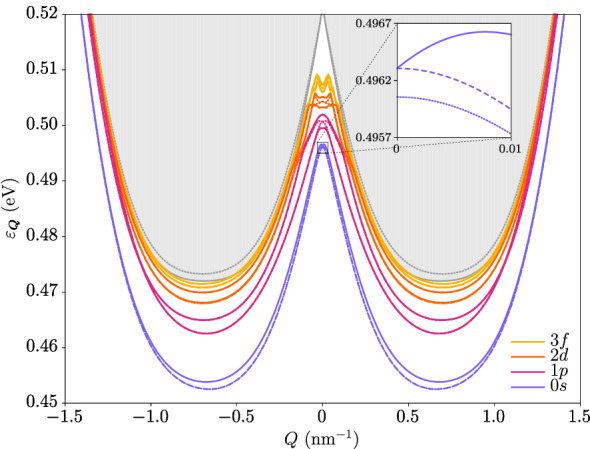
Excitonic dispersion relation for the Rytova–Keldysh potential. Shown are the exciton eigenstates $| \varvec{Q}; 0_{\pm } \rangle$ (dotted lines), $| \varvec{Q};2_{+} \rangle$ (solid lines), and $| \varvec{Q};2_{-} \rangle$ (dashed lines), with $\varvec{Q}$ the total exciton momentum. Different colors correspond to different values of *m* at $\varvec{Q} = \varvec{0}$ as indicated in the legend. The 1*s* state (not shown) has higher energy than the 1*p*, 2*d* and 3*f* states. The excitons $| \varvec{Q},2_{\pm } \rangle$ with opposite angular momenta are split in energy, with the higher state having angular momentum $m > 0$ and the lower state $m < 0$. The gray region at the top represents the electron-hole continuum, delimited by a solid gray line resulting from analytically minimizing the energy gap with respect to the relative electron-hole momentum in the absence of interactions. The gray dotted line represents the continuum threshold as calculated numerically including only angular momenta $|m| \le 3$ and tends to the solid line upon inclusion of higher values of |*m*|.

**Figure 5:**
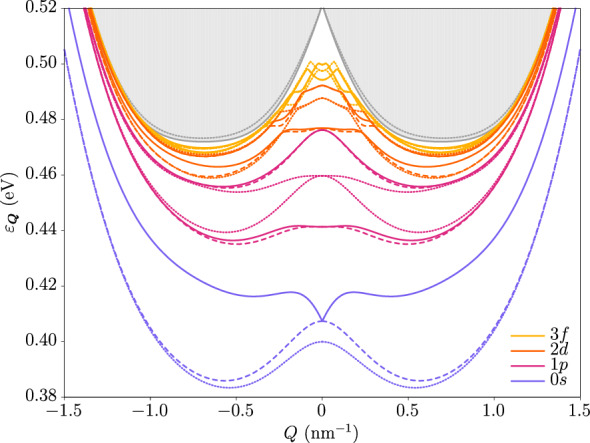
Excitonic dispersion relation for the Coulomb potential. All colors and line types are equivalent to their counterparts in Fig. 4.

**Figure 6:**
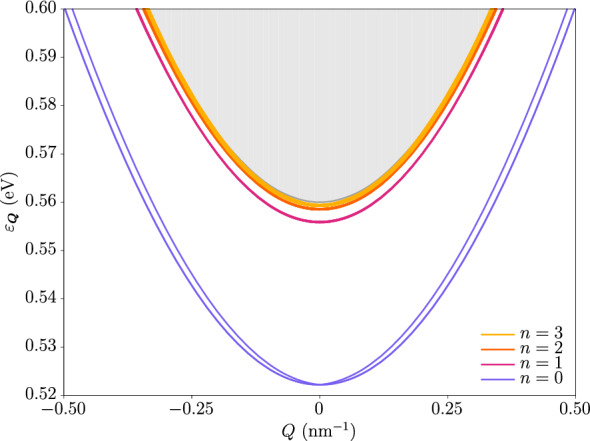
Excitonic dispersion relation for the Coulomb potential in the trivial regime. Here the different colors simply label the principal quantum number *n*. Up to numerical error, at $\varvec{Q} = \varvec{0}$ every state has a degeneracy $g_{n} = 4(2n + 1)$, consistent with the fact that there are four exciton families each having angular momentum $m \in \{-n, \dots , 0, \dots , n\}$. The binding energies at the origin follow the hydrogenic Rydberg series $\Delta _{n} \propto \big (n + \frac{1}{2}\big )^{-2}$. This plot has been obtained by using the same parameters for the underlying single-particle model but changing the sign of $B_{2}$, which produces a zero Chern number for the electrons and holes (see the “Methods” section).

We have solved the associated Bethe-Salpeter equation with both the Rytova–Keldysh potential and the two-dimensional Coulomb potential after neglecting the effects of the quantum confinement in the *z*-direction. The former is often used to approximate the long-distance behavior of the microscopic Keldysh potential^48^. We find that only the Rytova–Keldysh interaction is compatible with the neglect of quantum confinement, so unless otherwise specified all results correspond to those obtained with this potential. Figure 4 shows the dispersion relations of all four excitonic ground states, and also of several excited states that have a nonzero angular momentum at $\varvec{Q} = \varvec{0}$. The same is shown in Fig. 5 for the Coulomb interaction, whose weaker nature allows us to better visualize the features of the band structure that are qualitatively independent of the interaction details due to the robustness of the topology. Notice the difference in behavior of the energy between the $| \varvec{Q};2_\pm ;0,0 \rangle$ and $| \varvec{Q};0_\pm ;0,0 \rangle$ doublets around zero momentum, as the former linearly splits off. This effect is heavily influenced by the topology, as we have checked that the dispersions become strongly parabolic in the absence of a band inversion. This can be seen in Fig. 6, where we have plotted the exciton dispersion for the same underlying single-particle model but this time in the trivial regime. It is apparent that the nonanalytic mode is barely noticeable for all states. Interestingly, in fact, in this case the spectrum at the origin is fully hydrogen-like, as we obtain the Rydberg series for the binding energies $\Delta _{n} \propto \big (n + \frac{1}{2}\big )^{-2}$ and the usual hydrogenic degeneracies at zero exciton momentum.

The linear dispersion may be analytically understood by expanding the effective exchange potential around the origin of $\varvec{Q}$, which is further analyzed in Ref.^49^. Moreover, the effective $2 \times 2$ Hamiltonian for the $| \varvec{Q};2_\pm ;0,0 \rangle$ chiral doublet has been discussed previously^47,50,51^ and is further studied below. Going back to the topological regime, the energy of the $| \varvec{Q};0_\pm ;0,0 \rangle$ doublet follows a quadratic dispersion both at small and large momenta, with a crossover taking place around the minimum of the electron-hole continuum, as the inversion of the bands after that has less of an effect.

Interestingly, the states $| \varvec{Q};2_{\pm } \rangle$ with opposite angular momenta $+m$ and $-m$ are split in energy, with the former having higher energy than that of the latter, as seen in the figures for the solid and dashed lines. This is a consequence of the Berry curvature within the nanosheet^39^, which provides an anomalous contribution to the single-particle velocity that is only present in the subspace spanned by the $| \varvec{Q};+,+ \rangle$ and $| \varvec{Q};-,- \rangle$ states. Our results agree qualitatively with previous works that perturbatively incorporate the effects of the Berry curvature^37–42^. However, numerical discrepancies are expected between these references and the present work, as the former all consider the effective-mass approximation. This is not appropriate in our system due to the band inversion, which prevents us from decoupling the relative and center-of-mass motions. Consequently, our exciton spectra at zero momentum deviate from the well-known Rydberg series in the inverted regime.

Also, the energies of the higher excited states closely follow the electron-hole continuum, as the excitons are more delocalized in real space and their wave functions become those of a separate electron-hole pair. An important feature of the Rytova–Keldysh spectrum is that all excitons are strongly indirect, which leads to long lifetimes due to the strongly reduced radiative recombination rate. As revealed by the shape of the electron-hole continuum, this is a direct consequence of the band inversion of the underlying single-particle bands. Note, however, that in this work both the electron and the hole are taken to reside in the same material layer and are thus still direct in real space.

**Figure 7:**
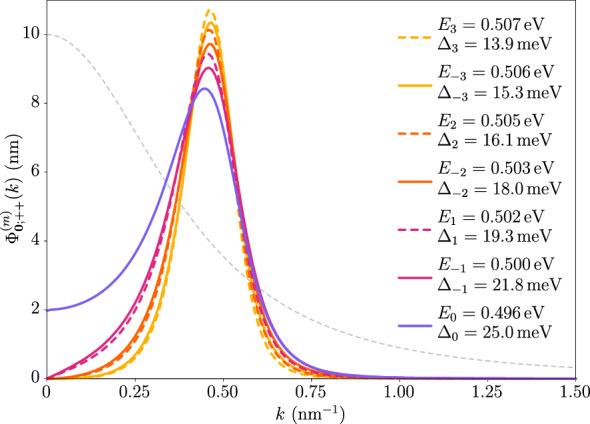
Magnitudes of momentum-space exciton wave functions. These correspond to the zero-momentum states $| \varvec{0};+,+;n,m \rangle$ and $| \varvec{0};-,-;n,-m \rangle$ and are shown for several values of *m* as ordered in Fig. 4 and the first available *n*. The wave functions themselves are obtained by multiplying the magnitude by the corresponding phase $e^{i m \phi _{\varvec{k}}}$. The wave functions have a maximum around the momentum at which the energy gap presents a minimum. In particular, the wave function in the *s*-wave case (purple line) significantly differs from that obtained from the hydrogen problem, which is proportional to $((a_0 k)^2 + 1)^{-3/2}$ (gray dashed line). Here we have set $a_{0} = 10/\sqrt{8 \pi }\,\hbox{nm} \simeq {1.99}\,\hbox {nm}$, which is suitable for comparison. The values $E_m$ shown in the figure correspond to the excitonic eigenenergies for the same states. The quantities $\Delta _m$ are the binding energies of each state, that is, the difference between the electron-hole continuum and $E_m$.

**Figure 8:**
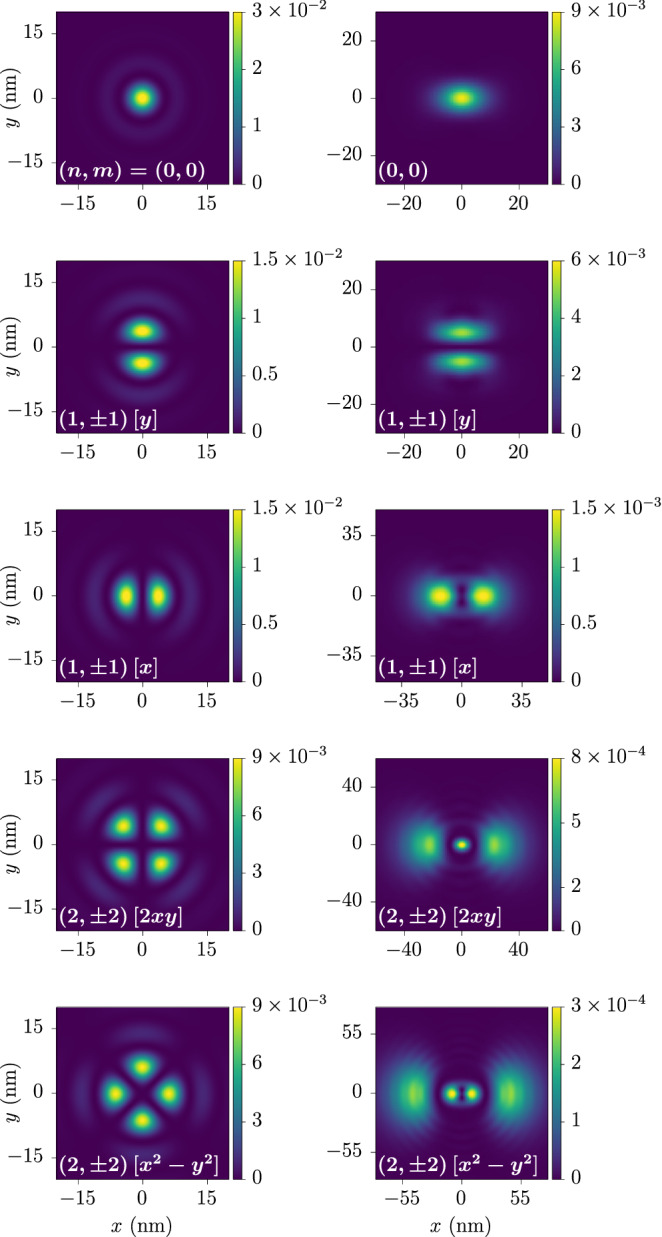
Relative real-space probability densities of the exciton eigenstates $|\varvec{Q}; 0_{\pm }; n, m \rangle$. The rows correspond, from top to bottom, to the first five dotted-line states of Fig. 4. In the left column, $\varvec{Q} = \varvec{0}$ and we take appropriate linear combinations resulting in hydrogen-like orbitals. The right column corresponds to $\varvec{Q}$ along the *x*-direction, with $Q = {0.7}\,{\hbox {nm}^{-1}}$. The values of *n* and *m* are indicated in each plot, as well as the orbital name in the standard notation.

**Figure 9:**
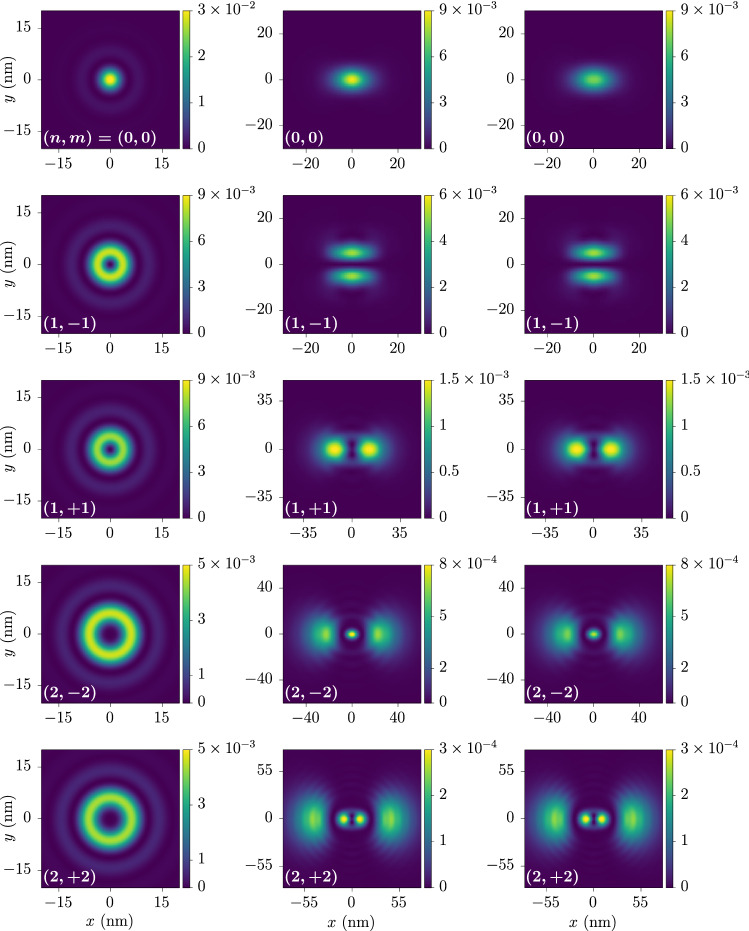
Relative real-space probability densities of the exciton eigenstates $| \varvec{Q}; 2_{\pm }; n, m \rangle$. The rows correspond, from top to bottom, to the first five solid- and dashed-line states of Fig. 4. More precisely, the states shown are $| \varvec{0}; 2_{\pm }; n, m \rangle$ (left column), $| \varvec{Q}; 2_{-}; n, m \rangle$ (middle column), and $| \varvec{Q}; 2_{+}; n, m \rangle$ (right column). The values of *n* and *m* are indicated in each plot. We have set $\varvec{Q}$ along the *x*-direction with $Q = {0.7}\,{\hbox {nm}^{-1}}$.

Figure 7 shows the relative exciton wave function of the states $| \varvec{Q};2_{\pm } \rangle$ for the ground state and several excited states from Fig. 4 at $\varvec{Q} = \varvec{0}$. Note the significantly different behavior of the ground-state wave function compared to that obtained from the hydrogen problem, which is proportional to ${((a_0 k)^2 + 1)^{-3/2}}$. Furthermore, Figs. 8 and 9 show the relative real-space probability density of the low-lying states $| \varvec{Q}; 0_{\pm } \rangle$ and $| \varvec{Q}; 2_{\pm } \rangle$, respectively. For the states $| \varvec{Q};0_{\pm } \rangle$ at $\varvec{Q} = \varvec{0}$ we exploit their opposite-angular-momentum degeneracy to obtain linear superpositions resulting in hydrogen-like orbitals shown in the left column, and note how these become deformed in the direction of a nonzero exciton momentum in the right column due to the breaking of the rotational symmetry. In the case of the states $| \varvec{Q}; 2_{\pm } \rangle$, the splitting between states with opposite *m* at $\varvec{Q} = \varvec{0}$ prevents us from writing down such orbitals, so the first column shows a rotationally invariant probability density. Nevertheless, a nonzero exciton momentum breaks again this rotational symmetry, and the wave functions develop lobes in either the transversal or the longitudinal direction as seen in the second and third columns.

### Optical properties

We have also derived selection rules for circularly polarized light in the *xy*-plane moving in the positive *z*-direction at $\varvec{Q} = \varvec{0}$, where the exchange interaction vanishes and the exciton families $| \varvec{Q};+,+ \rangle$ and $| \varvec{Q};-,- \rangle$ become uncoupled. For left-circularly polarized light (with angular momentum ${m_{\gamma } = +1}$) we find that the excitons $| \varvec{0}; +,+; n, 0 \rangle$ and $| \varvec{0}; -,-; n, {+}2 \rangle$ are bright, whereas the rest are dark. On the other hand, for right-circularly polarized light ${(m_{\gamma } = {-}1)}$ the only optically active excitons are $| \varvec{0}; -,-; n, 0 \rangle$ and $| \varvec{0}; +,+; n, {-}2 \rangle$. Note that these results combined are in accord with the time-reversal symmetry. By contrast, the excitons $| \varvec{0};+,- \rangle$ and $| \varvec{0};-,+ \rangle$ are all dark irrespective of their angular momentum. These results greatly differ from the situation in ordinary semiconductors, where only the *s*-wave singlet is bright.

### Effective exciton Hamiltonian

For small momenta, the behavior of each pair of $| \varvec{Q};+,+ \rangle$ and $| \varvec{Q};-,- \rangle$ excitons can be understood by means of an effective $2 \times 2$ model. This model has been presented before^47,50,51^ and is given by

4 $$\begin{aligned} H_{X}^{\text {eff}}({\varvec{Q}}) = \bigg (\hbar \omega _{X} + \frac{\hbar ^2 Q^{2}}{2M_{X}}\bigg ) + \mathcal {J}_{X}({\varvec{Q}}) \begin{bmatrix} 1 &{} e^{-2i\phi _{{\varvec{Q}}}} \\ e^{2i\phi _{{\varvec{Q}}}} &{} 1 \end{bmatrix} ~, \end{aligned}$$

where $X = (n,m)$ labels the particular exciton doublet. Here, $\hbar \omega _{X}$ is the energy of the doublet at zero momentum, $M_{X}$ is an effective mass, and

5 $$\begin{aligned} \mathcal {J}_{X}({\varvec{Q}}) = {\left\{ \begin{array}{ll} 0 + \mathcal {O}(Q^{3}) &{} \text {if}\; m\; \text {is odd} ~, \\ J_{X} Q + \frac{\hbar ^{2} Q^{2}}{2 M'_{X}} + \mathcal {O}(Q^{3}) &{} \text {if}\; m\; \text {is even} ~. \end{array}\right. } \end{aligned}$$

According to Eq. (5), the states with odd angular momentum at $\varvec{Q} = \varvec{0}$ should remain degenerate for small $\varvec{Q}$, which is indeed the case in Figs. 4 and 5. Note that this degeneracy is broken to third order in *Q*, but this effect is not captured by our perturbative scheme. On the other hand, the states with even angular momentum may split into a linear mode and a quadratic mode, as is the case for the lowest *s*-wave and *d*-wave states in the figures. This effect is expected to be most important for *s*-wave excitons, as the lowest-order contribution to the splitting parameter $J_{X}$ is proportional to $|\Phi _{\varvec{Q}}(\varvec{r} = \varvec{0})|^{2}$, which is nonzero only for $m = 0$.

This effective model is time-reversal-symmetric, and thus one question to ask is what may happen upon breaking of this antiunitary symmetry. This can be accomplished by the inclusion of a small Zeeman-like perturbation $\Delta \sigma _{z}$. For the upper and lower bands of *s*-wave exciton doublets one then finds Berry curvatures

6 $$\begin{aligned} \Omega _{xy}^{\pm }(\varvec{Q}) = \pm 4 \Delta |M'| \frac{Q (JM'+Q) (2JM'+Q)}{\big [Q^{2}(2JM'+Q)^{2}+(2M' \Delta )^{2}\big ]^{3/2}} ~ . \end{aligned}$$

Integration leads to Chern numbers $\mathcal {C}_{\pm } = \pm {\text {sgn}} \Delta$, and thus the bands are topological. By the bulk-boundary correspondence, this may lead to topological boundary states of chiral excitons under suitable conditions described in the following section.

## Discussion

Our results show that, in principle, the topology of the conduction and valence bands is indeed inherited by the Wannier excitons, as the exciton wave function contains electron and hole pseudospin textures with a topologically nontrivial winding. This is in particular true for the excitonic basis states that diagonalize the Wannier problem with only the direct interaction included. As the exchange interaction couples these states the physical picture complicates somewhat, but the nontrivial pseudospin winding remains. Furthermore, the bare two-dimensional interaction is heavily modified by the topological band structure. For the excitonic ground state, this ultimately results in a nonchiral doublet with quadratic dispersion relation at low momenta, and a chiral doublet with one linearly dispersing mode and one quadratic mode. Breaking the time-reversal symmetry of these bands with a Zeeman-like term reveals the underlying nontrivial topology of these bands. Since our work does not impose many restrictions on the model parameters, we expect that it remains valid for other similar topological materials such as Bi_2_Te_3_ and Sb_2_Se_3_ by using the appropriate parameter values.

A future direction of research is to consider the motion of the excitons under the effects of lattice strain, which yields a confining potential in real space. In this scenario we expect that the nontrivial Berry curvature, which constitutes a momentum-space magnetic field, will give rise to an anomalous Hall effect^52,53^. Experimentally, we want to resolve the linear dispersion in the chiral doublet, which would be a first step towards the observation of the topological properties of excitons. This is in principle possible by means of terahertz spectroscopy, as the details of the dispersion affect the chemical equilibrium between excitons and free charges in such pump-probe experiments^54–56^. We also expect the polarizability of the excitons to be strongly affected by their topology, which can again be observed in terahertz conductivity measurements. In particular, the polarizability of the topological *s*-wave excitons should be significantly reduced with respect to that of their trivial counterparts due to their modified relative wave function. Another interesting feature of the obtained excitonic band structure is that it is indirect as a consequence of the band inversion of the single-particle Hamiltonian, which leads to a greatly reduced rate of radiative recombination processes and thus to long-lived excitons.

The selection rules we have derived can be understood by noting that the eigenstates $| \chi ^{\mu }_{\varvec{k}} \rangle$ are also eigenstates of the total angular momentum operator $j_{z} = l_{z} + \frac{1}{2} s_{z}$, where $s_{z}$ is the Pauli matrix acting on the spin part of the single-particle basis and $l_{z} = -i \partial _{\phi _{\varvec{k}}}$. Consequently, the single-particle states $| \chi ^{c,s}_{\varvec{k}} \rangle$ and $| \chi ^{v,t}_{\varvec{k}} \rangle$ have angular momenta $\frac{s}{2}$ and ${-}\frac{t}{2}$, respectively. By noting that the angular momentum of a hole is opposite to that of the destroyed valence electron, one finds that an exciton can couple to a left-circular photon if its relative angular momentum is 0 and the underlying electron and hole lie in the bands with $s = t = +1$, or if its relative angular momentum is $+2$ and the particles lie in the bands with $s = t = -1$, as we have seen. The argument follows analogously for right-circular photons.

By virtue of the topological nature of its bands, the effective exciton Hamiltonian can potentially host topologically protected exciton states at the edges of a finite sample. The Zeeman-like term $\Delta \sigma _{z}$ is necessary to open up a direct gap at the origin of $\varvec{Q}$. The resulting long-wavelength model can be diagonalized to obtain boundary modes at the interface of two regions with opposite $\Delta$, as done in Ref.^57^. However, we still require a *global* topological gap^58^. This is not present naturally in our model, as it is clear from Figs. 4 and 5 that the two exciton bands of the same doublet have the same asymptotic behavior for large *Q*. Such a gap can be achieved via a periodic exciton potential that introduces band backfolding at an appropriate nonzero value of $Q_{x}$ or $Q_{y}$, so as to introduce a Brillouin zone for the component of the total exciton momentum perpendicular to the edge. Possibilities for the realization of such a potential include the application of lattice strain, as already mentioned above, or the use of surface acoustic waves^59,60^. The properties of such edge states clearly warrant a thorough analysis that lies outside the scope of this article, but we intend to investigate them next.

We briefly comment on the term $\Delta \sigma _{z}$, as it has been introduced by hand in the effective Hamiltonian. This is necessary because the underlying BHZ model is time-reversal symmetric and thus by itself will not give rise to such a term. However, the topological properties only depend on its sign, not on its magnitude, and emerge no matter how small $\Delta$ may be as long as it is nonzero. We are therefore allowed to add it to the effective model as a perturbation. Experimentally, this term may be realized by a time-reversal-breaking perturbation such as a small magnetic field, via contact to a thin magnetized layer, or with the injection of a small concentration of magnetic impurities. Furthermore, even though the topological properties imbued by $\Delta$ may seem independent of the state of the underlying BHZ Hamiltonian, one must keep in mind that the effective model for the chiral excitons arises only in the topological regime. Consequently, both ingredients are required for the emergence of topological excitons.

Finally, the (top and bottom) surface electronic states, which have been neglected in this work, may contribute to several aspects that will be analyzed in a follow-up article. First, by screening the interaction between bulk electrons and holes, which may be treated as static screening of the Coulomb or Rytova–Keldysh potentials. We have in first instance neglected this effect because of the reduced density of states around the Dirac cone, to which the screening length will be inversely proportional, at least in the Thomas-Fermi regime. Note that we are always considering single-exciton excitations in the material, so that electron-electron correlation effects are effectively incorporated in the parameters of the band structure, for instance via GW corrections. Second, by causing a nonzero transition probability from bulk states to surface states mediated by surface plasmons, which thus in principle provide a mechanism for surface electron-hole decay of bulk excitons. The effects of both the screening and exciton decay will be analyzed perturbatively to verify the correctness of our results, as it depends on the magnitude of the energy shifts and spectral broadenings induced by these phenomena. Away from zero chemical potential, we expect quasi-2D nanosheets of Bi_2_Se_3_ with around 6 QLs to provide a promising platform for exciton-plasmonics, which will constitute the main topic of the sequel. Third, by a contribution to the complex conductivity measured in pump-probe terahertz conductivity experiments, which may be modelled as a separate contribution from that of the bulk states, as similarly done for unbound charges in Ref.^54^. However, we expect the contribution from the surface states to the absorption to be small compared to that of the bulk states, as the oscillator strength of the latter is much larger than that of the former.

## Methods

### Band structure of Bi_2_Se_3_ nanosheets

Our starting point is the $\varvec{k}\varvec{\cdot }\varvec{p}$ Hamiltonian derived previously^61,62^ for modeling three-dimensional Bi_2_Se_3_ and Bi_2_Te_3_ around the $\Gamma$ point in the Brillouin zone. To account for the quantization in the *z*-direction in a nanosheet geometry of thickness $L_{z}$, we solve the model at the two-dimensional $\Gamma$ point ${(k_{x} = k_{y} = 0)}$ with the substitution ${k_{z} \rightarrow -i \partial _{z}}$ and hard-wall boundary conditions as done before^63,64^. Everywhere in our numerics we consider ${L_{z} = {6}\,{\hbox {nm}}}$, which in the case of Bi_2_Se_3_ is sufficiently large for the single-particle Dirac states on the opposite surfaces not to be gapped out by tunneling processes and the nontrivial topology of the bulk to survive^65–67^, but also sufficiently small for bulk electrons and holes to still behave as in two dimensions. We then project the 3D Hamiltonian onto the energetically highest-lying valence and lowest-lying conduction subbands in order to integrate out the *z*-dependence. Hence, we assume that the relevant low-energy physics of individual particles is confined to this subspace, which has been verified *a posteriori* by comparing the obtained exciton binding energies to the energy splitting of the bulk subbands due to the confinement in the *z*-direction.

Ultimately, after projecting the three-dimensional $\varvec{k}\varvec{\cdot }\varvec{p}$ Hamiltonian onto the single-particle ground states of the 3D model, we obtain our desired nanosheet Hamiltonian. Written in terms of Pauli matrices in spin and orbital space, $\varvec{s}$ and $\varvec{\tau }$ respectively, it is given by the $4\times 4$ matrix

7 $$\begin{aligned} H_0(\varvec{k}) = \epsilon _0(\varvec{k}) + M(\varvec{k})\tau _z + A_2(k_x s_x + k_y s_y) \tau _x ~ , \end{aligned}$$

where $\varvec{k} \equiv (k_{x}, k_{y})$ is the in-plane momentum, $\epsilon _0(\varvec{k}) \equiv E + D_2 (k_x^2 + k_y^2)$, and $M(\varvec{k}) \equiv M - B_2 (k_x^2 + k_y^2)$. Note that products of matrices in different spaces, e.g., $s_x \tau _x$, are Kronecker products and not matrix products, and that identity matrices are implied. Our effective two-dimensional Hamiltonian is equivalent to that of the BHZ model after a suitable unitary transformation. Furthermore, as expressed in Eq. (7) it has the same form as the three-dimensional Bi_2_Se_3_ Hamiltonian with $k_{z} = 0$ and some renormalized values of the parameters with respect to those given in Ref.^61^ However, the basis in which it is expressed differs from that of the 3D model, since the original $\textrm{Bi}^{+}$ and $\textrm{Se}^{-}$ orbitals have hybridized in the corresponding eigenstates. We denote these hybridized orbitals by $\mathrm {Bi'}^{+}$ and $\mathrm {Se'}^{-}$. The renormalized values of the parameters, with which all of our numerical results have been obtained, are $A_2 = {0.41}\,{\hbox {eV nm}}$, $M = {0.28}\,{\hbox {eV}}$, $B_2 = {0.473}\,\hbox {eV}\,{\hbox {nm}^2}$, $E = {-\,0.0012}\,{\hbox {eV}}$, and $D_2 = {0.202}\,\hbox {eV}\,{\hbox {nm}^2}$.

Diagonalization of $H_{0}(\varvec{k})$ leads to the conduction and valence band energies

8 $$\begin{aligned} \epsilon _{c,v}(\varvec{k}) = \epsilon _0(\varvec{k}) \pm \sqrt{M(\varvec{k})^2 + A_2^2(k_x^2+k_y^2)} ~, \end{aligned}$$

as well as to the topologically nontrivial single-particle states that we use throughout in our description of excitons. As a consequence of time-reversal symmetry in combination with inversion symmetry, the Hamiltonian does not couple the two subspaces $\{ | \mathrm {Bi'}^{+};\uparrow \rangle ,| \mathrm {Se'}^{-};\downarrow \rangle \}$ and $\{ | \mathrm {Bi'}^{+};\downarrow \rangle ,| \mathrm {Se'}^{-};\uparrow \rangle \}$, and the conduction and valence bands are each two-fold degenerate. The corresponding eigenstates are labeled by their so-called spin-orbit parity, defined as the eigenvalue of the operator $s_{z} \tau _{z}$ that commutes with $H_{0}(\varvec{k})$.

We next introduce the vector of pseudospin operators $\varvec{\Gamma } \equiv (s_x\tau _x,\,s_y\tau _x,\,\tau _z)$, which are used to rewrite Eq. (7) as $H_0(\varvec{k}) = \epsilon _0(\varvec{k}) + \varvec{d}(\varvec{k}) \varvec{\cdot } \varvec{\Gamma }$, with $\varvec{d}(\varvec{k}) \equiv (A_2 k_x, \, A_2 k_y, \, M(\varvec{k}))$. The nontrivial topology of the Hamiltonian is now very explicit in this form, since $\varvec{d}(\varvec{k})$ is a skyrmion texture in the momentum plane $(k_x, k_y)$ and the operator $\varvec{\Gamma }$ reduces exactly to the three Pauli matrices in the uncoupled even and odd spin-orbit-parity subspaces. Therefore, the expectation value of the pseudospin as a function of $\varvec{k}$ follows the winding of $\varvec{d}(\varvec{k})$ and the winding number of the latter is up to a possible sign equal to the Chern number of the conduction and valence bands. The winding number for the subspace with spin-orbit parity $s \in \{+,-\}$ is $\frac{s}{2}({\text {sgn}} M + {\text {sgn}} B_{2})$, which is nontrivial when *M* and $B_{2}$ have the same sign.

### Exciton basis and wave functions

The conduction and valence states are $\langle \varvec{x};a | \varvec{k};\mu \rangle = (e^{i \varvec{k}\varvec{\cdot }\varvec{x}}/\sqrt{V}) \langle a | \chi _{\varvec{k}}^{\mu } \rangle$, where $V = L_x L_y$, the states $| a \rangle$ denote our four-dimensional combined spin and orbital basis states, and $| \chi ^{\mu }_{\varvec{k}} \rangle$ with $\mu \in \{c,v\} \times \{+,-\}$ are the eigenstates of the Hamiltonian in Eq. (7). Note that the latter are not periodic due to the fact that our Hamiltonian is derived from $\varvec{k} \varvec{\cdot } \varvec{p}$ theory and only accurately models the band structure around the $\Gamma$ point. An exciton state $X \equiv \{\varvec{Q},s,t\}$ is labeled by the total momentum $\varvec{Q}$ and the spin-orbit-parities *s* and *t* of the electron and hole bands, respectively, as well as by an additional set of ro-vibrational quantum numbers that we introduce later. Such an exciton state is a bound state in the polarization^68^, which in second quantization is determined by the pair correlation function

9 $$\begin{aligned}&\big \langle {\hat{\psi }}_{a}(\varvec{x}_1) {\hat{\psi }}_{b}^\dagger (\varvec{x}_2)\big \rangle _{X} \nonumber \\&\quad = \sum _{\varvec{k}} \Phi ^{s,t}_{\varvec{Q},\varvec{k}} \langle \varvec{x}_1;a | \varvec{Q}/2 + \varvec{k};c,{s} \rangle \langle -\varvec{Q}/2 + \varvec{k}; v,{t} | \varvec{x}_2;b \rangle \nonumber \\&\quad = \frac{e^{i \varvec{Q} \varvec{\cdot } \varvec{R}}}{V} \sum _{\varvec{k}} \Phi ^{s,t}_{\varvec{Q},\varvec{k}} \, e^{i \varvec{k}\varvec{\cdot } (\varvec{x}_1 - \varvec{x}_2)} \langle a | \chi _{\varvec{Q}/2 + \varvec{k}}^{c,{s}} \rangle \langle \chi _{{-}\varvec{Q}/2 + \varvec{k}}^{v,{t}} | b \rangle ~, \end{aligned}$$

where $s,t \in \{+,-\}$ and $\varvec{R} \equiv (\varvec{x}_{1} + \varvec{x}_{2})/2$ is the position of the exciton. Here $\Phi ^{s,t}_{\varvec{Q},\varvec{k}}$ is the relative wave function of the exciton in momentum space. One may also perform a particle-hole transformation so that holes become positive-energy excitations. In terms of electrons and holes, the conduction states remain unchanged, $\langle a | \chi _{\varvec{q}}^{e,s} \rangle = \langle a | \chi _{\varvec{q}}^{c,s} \rangle$. By contrast, the hole states satisfy $\langle b | \chi _{-\varvec{q}}^{h,t} \rangle = \langle \chi _{\varvec{q}}^{v,t} | b \rangle$. In this picture the pair correlation function thus reads

10 $$\begin{aligned} \big \langle {\hat{\psi }}_{a}&(\varvec{x}_1){\hat{\psi }}_{b}(\varvec{x}_2)\big \rangle _{X} \nonumber \\&\quad = \frac{e^{i \varvec{Q} \varvec{\cdot } \varvec{R}}}{V} \sum _{\varvec{k}} \Phi ^{s,t}_{\varvec{Q},\varvec{k}} \, e^{i \varvec{k}\varvec{\cdot } (\varvec{x}_1 - \varvec{x}_2)} \langle a | \chi _{\varvec{Q}/2 + \varvec{k}}^{e,{s}} \rangle \langle b | \chi _{\varvec{Q}/2 - \varvec{k}}^{h,{t}} \rangle ~. \end{aligned}$$

If desired we can then also obtain a first-quantized wave function, which is given by

11 $$\begin{aligned} \Psi ^{X}_{ab}(\varvec{x}_1, \varvec{x}_2) =\,&\frac{1}{V} \frac{e^{i \varvec{Q} \varvec{\cdot } \varvec{R}}}{\sqrt{V}} \sum _{\varvec{k}} e^{i \varvec{k}\varvec{\cdot } (\varvec{x}_1 - \varvec{x}_2)} \nonumber \\&\times \frac{1}{\sqrt{2}} \left( \Phi ^{s,t}_{\varvec{Q},\varvec{k}} {\langle a | \chi _{\varvec{Q}/2 + \varvec{k}}^{e,{s}} \rangle}_{1} {\langle b | \chi _{\varvec{Q}/2-\varvec{k}}^{h,{t}} \rangle }_{2} - \Phi ^{s,t}_{\varvec{Q},{-}\varvec{k}} {\langle b | \chi _{\varvec{Q}/2 + \varvec{k}}^{e,{s}} \rangle }_{2} {\langle a | \chi _{\varvec{Q}/2+\varvec{k}}^{h,{t}} \rangle }_{1} \right) ~. \end{aligned}$$

This is normalized provided that $\frac{1}{V} \sum _{\varvec{k}} |\Phi ^{s,t}_{\varvec{Q},\varvec{k}}|^{2} = 1$. Note that interchanging both particles, thus $\varvec{x}_{1} \leftrightarrow \varvec{x}_{2}$ and $(a,1) \leftrightarrow (b,2)$, results in an overall minus sign after we perform the transformation $\varvec{k} \rightarrow -\varvec{k}$.

The single-particle states, together with the relative momentum states ${| \varvec{k} \rangle }_{\textrm{rel}}$, describe the exciton states $| \varvec{Q};s,t \rangle$ fully in Dirac notation as

12 $$\begin{aligned} | \varvec{Q};s,t \rangle = \frac{1}{\sqrt{V}} | \varvec{Q} \rangle \sum _{\varvec{k}} \Phi ^{s,t}_{\varvec{Q},\varvec{k}} {| \varvec{k} \rangle }_{\textrm{rel}} {| \chi _{\varvec{Q}/2 + \varvec{k}}^{e,{s}} \rangle } | \chi _{\varvec{Q}/2-\varvec{k}}^{h,{t}} \rangle ~, \end{aligned}$$

where $\langle \varvec{R} | \varvec{Q} \rangle = e^{i\varvec{Q}\varvec{\cdot }{\varvec{R}}} / \sqrt{V}$. The four combinations of the spin-orbit-parity labels *s* and *t* lead to four distinct exciton basis states, similar to the singlet and triplet excitons in regular semiconductors. As explained in the next section, the states $| \varvec{Q};s,t \rangle$ in Eq. (12) are exact eigenstates of the Wannier problem with only the direct interaction included. This is in fact a much used approximation in the literature^43^, but in our case not sufficiently accurate as the exchange interaction in principle couples these states for $\varvec{Q} \ne \varvec{0}$. Nevertheless, the above set of states can still be considered as the most appropriate basis for the full excitonic problem. Further note that, as advanced, $| \varvec{Q};s,t \rangle$ more precisely stands for an entire family of ro-vibrational states which must be labeled by additional quantum numbers describing the different relative wave functions that solve the exciton problem.

The topology of these exciton basis states can now be intuitively understood, as in the single-electron case, from the dependence of the expectation value of the pseudospin $\varvec{\Gamma }_{e}(\varvec{Q})$ on the momentum $\varvec{Q}$, obtained from Eq. (12) as

13 $$\begin{aligned} \varvec{\Gamma }_{e}(\varvec{Q}) = \frac{1}{V} \sum _{\varvec{k}} |\Phi ^{s,t}_{\varvec{Q},\varvec{k}}|^2 \langle \chi _{\varvec{Q}/2+\varvec{k}}^{e,s} | \varvec{\Gamma } | \chi _{\varvec{Q}/2+\varvec{k}}^{e,s} \rangle ~, \end{aligned}$$

and similarly for the pseudospin of the hole.

To determine the topology of the states $| \varvec{Q};s,t \rangle$ mathematically more rigorously, we need to compute the winding number of the pseudospin. Note that for the moment we particularize to the case of a globally vanishing exchange interaction, in which case all states $| \varvec{Q};s,t \rangle$ are uncoupled and therefore have a well-defined Chern number. To access the topological properties we must compute the Berry connection^8–12^, which in the excitonic case is given by a sum of three distinct terms, $\varvec{A}_{s,t}(\varvec{Q}) = \varvec{A}^{(e)}_{s,t}(\varvec{Q}) + \varvec{A}^{(h)}_{s,t}(\varvec{Q}) + \varvec{A}^{(\Phi )}_{s,t}(\varvec{Q})$. These read

14 $$\begin{aligned} \varvec{A}^{(e)}_{s,t}(\varvec{Q})&= -\frac{i}{V} \sum _{\varvec{k}} |\Phi ^{s,t}_{\varvec{Q},\varvec{k}}|^2 \langle \chi _{\varvec{Q}/2+\varvec{k}}^{e,s} | \varvec{\nabla }_{\varvec{Q}} | \chi _{\varvec{Q}/2+\varvec{k}}^{e,s} \rangle ~, \end{aligned}$$

15 $$\begin{aligned} \varvec{A}^{(h)}_{s,t}(\varvec{Q})&= -\frac{i}{V} \sum _{\varvec{k}} |\Phi ^{s,t}_{\varvec{Q},\varvec{k}}|^2 \langle \chi _{\varvec{Q}/2-\varvec{k}}^{h,t} | \varvec{\nabla }_{\varvec{Q}} | \chi _{\varvec{Q}/2-\varvec{k}}^{h,t} \rangle ~, \end{aligned}$$

16 $$\begin{aligned} \varvec{A}^{(\Phi )}_{s,t}(\varvec{Q})&= -\frac{i}{V} \sum _{\varvec{k}} (\Phi ^{s,t}_{\varvec{Q},\varvec{k}})^{*} \, \varvec{\nabla }_{\varvec{Q}} \Phi ^{s,t}_{\varvec{Q},\varvec{k}} ~. \end{aligned}$$

Each of these terms contributes to the local Berry curvature or momentum-space magnetic field through $\varvec{B}^{(i)}_{s,t}(\varvec{Q}) = \varvec{\nabla }_{\varvec{Q}} \times \varvec{A}^{(i)}_{s,t}(\varvec{Q})$, where $i \in \{e, h, \Phi\}$. Finally, the Berry curvature is connected to the desired winding or Chern number by

17 $$\begin{aligned} \mathcal {C} = \frac{1}{2\pi } \int \textrm{d}^{2} \varvec{Q} \varvec{\cdot } \varvec{B}(\varvec{Q}) = \frac{1}{2 \pi } \oint \textrm{d} \varvec{Q} \varvec{\cdot } \varvec{A}(\varvec{Q}) ~, \end{aligned}$$

where the first surface integral is performed over the first Brillouin zone, which in our continuum model amounts to integration over the infinite momentum space, and the equivalent line integral is performed along a contour with $Q \rightarrow \infty$. It is clear that the terms $\varvec{B}^{(e)}_{s,t}(\varvec{Q})$ and $\varvec{B}^{(h)}_{s,t}(\varvec{Q})$ not only contain the direct weighted Berry curvature of the underlying single particles, but also an interference term between the single-particle Berry connection and the excitonic envelope wave function. However, the total contribution of these terms to the exciton Chern number is given only by the single-particle electron or hole Chern number. This can be shown, e.g., for the electron case, by first expanding $\langle \chi ^{e,s}_{\varvec{Q}/2 + \varvec{k}} | \varvec{\nabla }_{\varvec{Q}} | \chi ^{e,s}_{\varvec{Q}/2 + \varvec{k}} \rangle$ with respect to $\varvec{k}$ and noticing that $\varvec{\nabla }_{\varvec{k}} | \chi ^{e,s}_{\varvec{Q}/2 + \varvec{k}} \rangle \big |_{\varvec{k} = \varvec{0}} = 2 \varvec{\nabla }_{\varvec{Q}} | \chi ^{e,s}_{\varvec{Q}/2} \rangle$. In our model one then observes that all terms in the line integral decay faster with *Q* than the zeroth-order term $\langle \chi ^{e,s}_{\varvec{Q}/2} | \varvec{\nabla }_{\varvec{Q}} | \chi ^{e,s}_{\varvec{Q}/2} \rangle$ and thus vanish on the contour at infinity. Therefore,

18 $$\begin{aligned} \mathcal {C}_{e} = {-} \frac{i}{2 \pi } \oint \textrm{d} \varvec{Q} \varvec{\cdot } \bigg (\frac{1}{V} \sum _{\varvec{k}} |\Phi ^{s,t}_{\varvec{Q,\varvec{k}}}|^{2} \langle \chi ^{e,s}_{\varvec{Q}/2} | \varvec{\nabla }_{\varvec{Q}} | \chi ^{e,s}_{\varvec{Q}/2} \rangle \bigg ) ~, \end{aligned}$$

and the normalization condition $\frac{1}{V} \sum _{\varvec{k}} |\Phi ^{s,t}_{\varvec{Q,\varvec{k}}}|^{2} = 1$ may now be used. We are then left precisely with the expression for the free electron Chern number.

There can in principle be an additional contribution to the Chern number due to the envelope wave function itself, given by $\mathcal {C}_{\Phi } = \frac{1}{2 \pi } \oint \textrm{d} \varvec{Q} \varvec{\cdot } \varvec{A}^{(\Phi )}_{s,t}(\varvec{Q})$. However, this can be shown to vanish here by analyzing the winding of the direct interaction around the origin of $\varvec{Q}$. This winding is trivial, meaning that the potential does not pick up a phase as one circles around this point. That is, $V^{\textrm{D}}(\varvec{Q}; k, \phi _{\varvec{k}}, k', \phi _{\varvec{k}'}) = {V^{\textrm{D}}(Q \hat{\varvec{x}}; k, \phi _{\varvec{k}} - \phi _{\varvec{Q}}, k', \phi _{\varvec{k}'} - \phi _{\varvec{Q}})}$, where $\phi _{\varvec{q}}$ is the angle between the momentum $\varvec{q}$ and the *x*-axis. A straightforward analysis of the exciton eigenproblem then shows that we can choose a gauge such that $\Phi ^{s,t}_{\varvec{Q}}(k, \phi _{\varvec{k}}) = \Phi ^{s,t}_{Q \hat{\varvec{x}}}(k, \phi _{\varvec{k}} - \phi _{\varvec{Q}})$. Observing that the system enjoys reflection symmetry with respect to the *x*-axis when $\varvec{Q} = Q \hat{\varvec{x}}$, this may now be used to verify that $\mathcal {C}_{\Phi } = 0$.

We thus conclude that all global topological properties of the excitons are introduced by the electron and hole states $| \chi _{\varvec{Q}/2 + \varvec{k}}^{e,{s}} \rangle$ and $| \chi _{\varvec{Q}/2-\varvec{k}}^{h,{t}} \rangle$. Hence, the total Chern number of the above exciton basis states is $\mathcal {C} = \mathcal {C}_{e} + \mathcal {C}_{h}$, which for our BHZ model becomes $\mathcal {C} = s + t$ by explicit calculation. Physically, this result can be most easily understood by the fact that the even and odd spin-orbit-parity subspaces are related by time-reversal symmetry and that the wave function for the hole is the complex conjugate of the electronic valence-band wave function, as we have seen. Note, however, that the local properties that influence for instance the electron and hole transport are quantified by the Berry curvature, and are thus still dependent on the precise shape of the relative wave functions and the interference terms.

We stress that the intuitive picture given above is only valid in the case of zero exchange interaction. As seen in the following section, when this is included the two subspaces with $s = t$ become coupled together and cannot be treated individually. The Chern number is then technically not well-defined since the time-reversal symmetry protects the degeneracy at $\varvec{Q} = \varvec{0}$. However, the nontrivial winding caused by the underlying Chern numbers remains, and as a result the full excitonic eigenstates still possess a chirality of $\pm 2$. This is crucially dependent on the fact that the exchange interaction that couples the two distinct subspaces winds nontrivially around the origin of $\varvec{Q}$, transforming instead as ${V^{\textrm{X}}(\varvec{Q}; k, \phi _{\varvec{k}}, k', \phi _{\varvec{k}'}) = e^{{-}2 i \phi _{\varvec{Q}}} V^{\textrm{X}}(Q \hat{\varvec{x}}; k, \phi _{\varvec{k}} - \phi _{\varvec{Q}}, k', \phi _{\varvec{k}'} - \phi _{\varvec{Q}})}$.

### Electron-hole interaction and Bethe–Salpeter equation

Having introduced the free part of the full Hamiltonian, we now proceed to discuss the electron-hole interaction potential that binds these particles together to form an exciton state. The interaction potential is $V_{s,t;s',t'}(\varvec{Q};\varvec{k},\varvec{k}') = V^{\textrm{D}}_{s,t;s',t'}(\varvec{Q};\varvec{k},\varvec{k}') - V^{\textrm{X}}_{s,t;s',t'}(\varvec{Q};\varvec{k},\varvec{k}')$, where $V^{{\text{D}}}$ and $V^{{\text{X}}}$ denote the direct and exchange interactions, respectively. These are given by

19 $$\begin{aligned} V^{\textrm{D}}_{s,t;s',t'}&(\varvec{Q};\varvec{k},\varvec{k}') =\delta _{s,s'} \delta _{t,t'} V(\varvec{k} - \varvec{k'}) \langle \chi _{\varvec{Q}/2+\varvec{k}}^{c,{s}} | \chi _{\varvec{Q}/2+\varvec{k}'}^{c,{s}} \rangle \langle \chi _{-\varvec{Q}/2+\varvec{k}'}^{v,{t}} | \chi _{-\varvec{Q}/2+\varvec{k}}^{v,{t}} \rangle ~, \end{aligned}$$

20 $$\begin{aligned} V^{\textrm{X}}_{s,t;s',t'}&(\varvec{Q};\varvec{k},\varvec{k}')=\delta _{s,t} \delta _{s',t'} V(\varvec{Q}) \langle \chi _{\varvec{Q}/2+\varvec{k}}^{c,{s}} | \chi _{-\varvec{Q}/2+\varvec{k}}^{v,s} \rangle \langle \chi _{-\varvec{Q}/2+\varvec{k}'}^{v,{s'}} | \chi _{\varvec{Q}/2+\varvec{k}'}^{c,s'} \rangle ~, \end{aligned}$$

where $V(\varvec{q})$ is the bare electrostatic potential within the nanosheet. A variational approach with the trial wave functions in the previous section leads to the Bethe-Salpeter equation^69,70^

21 $$\begin{aligned} \sum _{{\varvec{k}}', s', t'} \langle {\varvec{Q}}, {\varvec{k}}; s, t | H | {\varvec{Q}}, {\varvec{k}}'; s', t'\rangle \Phi _{{\varvec{Q}}, {\varvec{k}}'}^{s',t'} = {\varepsilon _{{\varvec{Q}}}} \Phi _{{\varvec{Q}}, {\varvec{k}}}^{s,t} ~. \end{aligned}$$

The matrix elements of the total Hamiltonian, including the electron-hole interaction, read

22 $$\begin{aligned} \langle {\varvec{Q}}, {\varvec{k}}; s, t | H | {\varvec{Q}}, {\varvec{k}}'; s', t'\rangle&= \big [\epsilon _{c}({\varvec{Q}}/2 + {\varvec{k}}) - \epsilon _{v}({\varvec{Q}}/2 - {\varvec{k}})\big ] \delta _{{\varvec{k}},{\varvec{k}}'} \delta _{s,s'} \delta _{t,t'} \nonumber \\&\quad + \frac{1}{V} \big [ V^{\textrm{D}} _{s,t;s',t'}({\varvec{Q}}; {\varvec{k}}, {\varvec{k}}') - V^{\textrm{X}}_{s,t;s',t'}({\varvec{Q}}; {\varvec{k}}, {\varvec{k}}') \big ] ~. \end{aligned}$$

We have solved this equation with both the bare two-dimensional Coulomb potential $V^{\textrm{C}}(\varvec{q})$ with the dielectric constant of the surrounding medium $\varepsilon _{s}$, and with the Rytova–Keldysh potential $V^{\textrm{RK}}(\varvec{q})$, which also takes into account the dielectric constant $\varepsilon _{d}$ of Bi_2_Se_3_ and is typically used in a nanosheet geometry. The explicit forms of these potentials in momentum space read

23 $$\begin{aligned} V^{\textrm{C}}(\varvec{q})&= {-}\frac{e^{2}}{2 \varepsilon _{0} \varepsilon _{s} q} ~, \end{aligned}$$

24 $$\begin{aligned} V^{\textrm{RK}}(\varvec{q})&= {-}\frac{e^{2}}{2 \varepsilon _{0} \varepsilon _{s}} \frac{1}{q(1 + r_{0}q)} ~, \end{aligned}$$

where $r_{0} = (\varepsilon _{d}/2 \varepsilon _{s}) L_{z}$ is a screening length that depends on the dielectric constants of Bi_2_Se_3_ and the surrounding environment. We have chosen the relative permittivity ${\varepsilon _{s} = 6}$, which is a typical low value for the environment^71^. The bulk dielectric constant has been set to ${\varepsilon _{d} = 28}$ in accord with recent first-principles studies of rhombohedral Bi_2_Se_3_ in the near-infrared region at 6 QLs^72,73^.

Our approach implies that we neglect the effects of quantum confinement on the classical electrostatic interaction. This is acceptable if the in-plane separation of the bound electron and hole is larger than the nanosheet thickness, as in this case the electric field lines will mostly lie in the surrounding environment. We find that the long-wavelength Coulomb interaction alone cannot accurately model excitons in bismuth selenide nanosheets because the resulting exciton diameters of the low-lying states are in fact smaller than the slab thickness, as seen in Table 1. Coincidentally, the Rytova–Keldysh interaction closely resembles the quantum-confined Keldysh potential at all momenta^74^, which is in any case guaranteed to accurately model the electrostatic interaction in this geometry. For this reason, it is not important which of these potentials enters Eqs. (19) and (20), and we have additionally checked that the difference in binding energies obtained with the Rytova–Keldysh and the quantum-confined Keldysh potentials are no larger than ${1}\,{\hbox {meV}}$, leading to errors lower than $10\%$. Note, however, that fully incorporating the quantum confinement would in principle lead to electrostatic interactions between subspaces with different spin-orbit parity due to the small overlap of the wave functions in the *z*-direction. This may have a small effect on the Rytova–Keldysh ground-state excitons, which are slightly smaller than the nanosheet thickness, but we note that the dielectric constant we have employed for the surrounding environment is relatively low and using a higher value would also lead to bigger excitons and thus mitigate this issue.

Additionally, we must compare the binding energies of the excitons with the splitting of the first two bulk subbands due to confinement, as we have neglected the subspaces of higher energy. Our approximation of projecting the 3D Hamiltonian onto the bulk subbands closest to the Fermi surface will be justified if the former is smaller than the latter. A binding energy larger than the bulk-subband splitting would imply the need to include the wave functions of higher excited states and thus effectively hinder the two-dimensional treatment of the problem. The splittings of the conduction and valence subbands for $L_{z} = {6}\,{\hbox {nm}}$ are found to be about ${61}\,{\hbox {meV}}$ and ${24}\,{\hbox {meV}}$, respectively, and the binding energies are given in Fig. 7. Except for the ground state, all states have a binding energy that is smaller than the relevant subband splittings. In the case of the ground state, the binding energy is only around ${1}\,{\hbox {meV}}$ larger than the valence-band splitting, so we do not expect that including the second subband will lead to significant modifications. We conclude that the Rytova–Keldysh potential is apt for our study of excitons in Bi_2_Se_3_ nanosheets, especially if we use a higher relative permittivity for the environment. Note, however, that the topology is neither affected by the values of the dielectric constants, nor by the precise form of the interaction potential.

**Table 1:** Mean exciton diameters of the zero-momentum states $| \varvec{0};+,+;0,m \rangle$ and $| \varvec{0};-,-;0,-m \rangle$ as obtained with the Coulomb and Rytova–Keldysh potentials with $\varepsilon _{s} = 6$ and $\varepsilon _{d} = 28$. In the case of the Coulomb potential the radii must be compared to the film thickness, which is $L_{z} = {6}\,{\hbox {nm}}$.

*m*		0	$-\,1$	$+\,1$	$-\,2$	$+\,2$	$-\,3$	$+\,3$
$\sqrt{\langle r^{2}\rangle }$ (nm)	$V^{\textrm{C}}$	2.74	4.21	5.32	6.33	7.61	9.04	9.76
	$V^{\textrm{RK}}$	5.64	7.02	7.51	8.72	9.13	10.57	10.89

Due to the orthogonality of the states in the different spin-orbit-parity subspaces, the exchange interaction $V^{\textrm{X}}$ only contributes when the spin-orbit parities of the electron and the hole are equal in both the initial and final states and when $\varvec{Q}\ne \varvec{0}$, as the inner products in Eq. (20) tend linearly to zero as a function of $\varvec{Q}$. Thus, specifically, the states $| \varvec{Q};+,- \rangle$ and $| \varvec{Q};-,+ \rangle$ are not coupled by the exchange interaction, whereas the states $| \varvec{Q};+,+ \rangle$ and $| \varvec{Q};-,- \rangle$ are. We can therefore solve the problem in the subspaces spanned by $| \varvec{Q};+,+ \rangle$ and $| \varvec{Q};-,- \rangle$ on the one hand, and by $| \varvec{Q};\pm ,\mp \rangle$ on the other hand. Since the interaction potentials are, up to complex conjugation, the same for the states $| \varvec{Q};+,- \rangle$ and $| \varvec{Q};-,+ \rangle$, the corresponding wave functions also only differ by complex conjugation, and both states are degenerate in energy for all $\varvec{Q}$. By contrast, the eigenstates spanned by $| \varvec{Q};+,+ \rangle$ and $| \varvec{Q};-,- \rangle$ are degenerate in energy only for $\varvec{Q} = \varvec{0}$.

### Derivation of optical properties

The oscillator strength of an exciton $| \varvec{0}; s, t; n, m \rangle$ reads^75,76^

25 $$\begin{aligned} f^{m, \hat{\varvec{e}}}_{s,t;n} = \frac{2}{\varepsilon ^{m}_{s,t;n}} \bigg |\int \textrm{d}^{2} k \, \Phi ^{(m)}_{\varvec{0}; s,t;n} \, e^{i m \phi _{\varvec{k}}} \, \hat{\varvec{e}} \varvec{\cdot } \langle \chi ^{v,t}_{\varvec{k}} | \varvec{v}(\varvec{k}) | \chi ^{c,s}_{\varvec{k}} \rangle \bigg |^{2} , \end{aligned}$$

where $\hat{\varvec{e}}$ is the Jones vector of the outgoing beam, $\varepsilon ^{m}_{s,t;n}$ is the energy of the zero-momentum exciton, and $\varvec{v}(\varvec{k})$ is the velocity operator. Since only interatomic transitions are expected to play a role in Bi_2_Se_3_, the velocity operator is well approximated by $\varvec{v}(\varvec{k}) \approx \varvec{\nabla }_{\!\varvec{k}} H_{0}(\varvec{k})$^77^. The Jones vectors of left- and right-circularly polarized light are $\hat{\varvec{e}}_{+}$ and $\hat{\varvec{e}}_{-}$, respectively, with $\hat{\varvec{e}}_{\pm } = \frac{1}{\sqrt{2}}(\hat{\varvec{x}} \pm i \hat{\varvec{y}})$. Computing the matrix elements that enter the above equation readily yields the reported results.

### Effective exciton Hamiltonian

The Hamiltonian of Eq. (4) can be obtained by computing the relevant matrix elements with the relative wave functions for $\varvec{Q} \rightarrow \varvec{0}$. The phase $\phi _{\varvec{Q}}$ must be handled with care in this limit by writing $\Phi _{\varvec{Q},\varvec{k}}^{s,t} \approx \Phi _{\varvec{0},|\varvec{k}|} \, e^{i m (\phi _{\varvec{k}} - \phi _{\varvec{Q}})}$ for excitons with angular momentum *m*. Another key point is that the symmetries of the potential matrix elements imply

26 $$\begin{aligned} \langle \varvec{Q}; +,+; n,m | {\hat{V}}^{X}| \varvec{Q};-,-;n,-m\rangle = e^{-2 i \phi _{\varvec{Q}}} \langle \varvec{Q}; +,+; n,m | {\hat{V}}^{X}| \varvec{Q};+,+;n,m\rangle ~, \end{aligned}$$

which results in the aforementioned coupling, This perturbation scheme is accurate to order $\varvec{Q}^{2}$, which nevertheless is not enough to break the degeneracy of the odd-*m* states away from nonzero $\varvec{Q}$. To capture this effect it is required to include corrections to the zero-momentum wave functions as well.

### Computational details

The Bethe-Salpeter equation has been solved with an independently developed MATLAB code that implements the discretization procedure of Ref.^78^ after rewriting all equations in terms of the dimensionless momentum $u = k L_{z}$ and keeping only contributions from angular momenta $|m| \le 3$. The integrals in the matrix elements have been performed up to a momentum cutoff $U = 10$ and with a discretization $\Delta u = 0.05$, and we have verified that the results do not depend on the cutoff by performing additional calculations up to $U = 40$. The long-wavelength divergence of the Coulomb potential has been regularized via an infrared cutoff $\Delta _{V} u$ chosen such that $\Delta _{V} u / \Delta u \approx 0.2262$, for which we have checked that the energies do not depend on $\Delta u$. If $\Delta _{V} u / \Delta u$ is chosen differently, a well-defined extrapolation of the exciton energy levels for $\Delta u \rightarrow 0$ can be done by using different values of the discretization.

In order to identify the angular momentum quantum numbers unambiguously after solving the Bethe-Salpeter equation, we choose a nonsingular gauge for the single-particle eigenstates that enter the direct and exchange potentials^39,75^. We have checked that with this choice the level ordering in the trivial regime reduces to that of the 2D hydrogen atom.

## Data Availability

The data that supports the findings of this study is available from the corresponding author upon reasonable request.

## Code availibility

The code that was employed to obtain the numerical results of this study is available from the corresponding author upon reasonable request.
